# Development of an IGF1R longevity variant mouse line using CRISPR/Cas9 genome editing

**DOI:** 10.15761/jtbr.1000121

**Published:** 2020-10-08

**Authors:** Yan Dou, Martin Darvas, Kavita Sharma, Julie Mathieu, John Morton, Heidi Tan, Carolina Soto-Palma, Luise A Angelini, Sara J McGowan, Laura J Niedernhofer, Yousin Suh, Paul D Robbins, Nir Barzilai, Warren C Ladiges

**Affiliations:** 1 Department of Comparative Medicine, School of Medicine, University of Washington, Seattle, WA, USA; 2 Department of Pathology, School of Medicine, University of Washington, Seattle, WA, USA; 3 Institute for Stem Cell and Regenerative Medicine, School of Medicine, University of Washington, Seattle, WA, USA; 4 Institute on the Biology of Aging and Metabolism, Department of Biochemistry, Molecular Biology and Biophysics, University of Minnesota, Minneapolis, MN, USA; 5 Department of Genetics and Development, Columbia University, New York, NY, USA; 6 Department of Medicine, Department of Genetics, Institute for Aging Research, Albert Einstein College of Medicine, New York, NY, USA

**Keywords:** human longevity, centenarians, IGF1R variants, CRISPR/Cas9 gene editing, Igf1r variant mouse model

## Abstract

An insulin-like growth factor-1 receptor (IGF1R) variant in exon 6 (Arg-407-His) in Ashkenazi Jewish centenarians was previously found to be associated with reduced IGF1R activity. To further study this longevity associated IGF1R variant, we generated a novel mouse line carrying the R407H variant in exon 6 of the *Igf1r* gene by employing CRISPR/Cas9 genome editing technology. Here, we show that the *Igf1r* gene can be edited in mouse embryos by zygotic electroporation of Cas9 protein and a single-guide RNAs together with a single stranded oligonucleotide donor containing the desired key nucleotide changes at the *Igf1r* locus. Sequence analysis of F0 and F1 mice following targeted editing demonstrated the robustness of this approach in mice using CRISPR/Cas9 directed homologous recombination (HDR). Western blot analysis indicates that mice heterozygous for the variant have a significant decrease in IGF1R phosphorylation in various tissues, including skeletal muscle, compared to wildtype. In addition, depletion of IGF1R signaling specifically in skeletal muscle of progeroid *Ercc1*^−/Δ^ mice resulted in extended health span and median lifespan providing the rationale for long term lifespan studies in *Igf1r* hR407H variant mice. This mouse line will be a valuable genetic tool to help determine the impact of IGF1R signaling on aging and longevity. The CRISPR editing approach represents a prototype for generating additional longevity associated gene variant mouse lines to study relevance to human exceptional longevity.

## Introduction

Loss of function in genes encoding components of insulin/insulin-like growth factor-1 (IGF1) signaling have been shown to extend lifespan in yeast, worms, flies, and mice [1]. While insulin mainly provides short-term effects that regulate metabolic pathways, IGF1 promotes chronic effects that determine cell fates, such as growth or differentiation of target cells, cell survival and maintenance of cell function [2]. The relevance of this regulatory network for exceptional longevity in humans remains unproven. Studies have reported on levels of IGF1 and related molecules in centenarians, but these lacked adequate controls. This shortcoming was overcome by establishing a cohort of Ashkenazi Jewish decent with extended longevity and their offspring, and age- and sex-matched controls without a family history of unusual longevity [3]. Using this group of individuals, variants in the *IGF1R* were found to be enriched in centenarians compared to controls. Immortalized lymphocytes from carriers of two nonsynonymous variants, A37T and R407H, showed significant reduction in IGF1R and its phosphorylation levels [3]. In a follow up study, engineered to express the two variants in *Igf1r*^−/−^ mouse embryonic fibroblasts (MEFs) also showed reduced phosphorylation of IGF1R [4].

These observations provided the rationale to further investigate the functional significance of the *IGF1R* variants in a genetically engineered mouse model and determine the impact on longevity. CRISPR/Cas9 genome editing is an established technology for generating genetic alterations in mice. However, it has not been applied to the development of mice expressing human longevity gene variants. In this report, we describe a CRISPR/Cas9 protocol to generate a mouse line with the human *IGF1R* variant R407H in exon 6, designated as *IGF1R*^hR407H^ mice. Our data demonstrate that *IGF1R*^hR407H^ mice have reduced IGF1R activity in multiple organs. In addition, a kinase-dead mutation in *Igf1r* extended the health span and median lifespan of progeroid *Ercc1*^−/Δ^ mice, consistent with lower IGF1R signaling in specific tissues contributing to the longevity and health of centenarians, which provides the rationale for lifespan studies in *IGF1R*^hR407H^ mice. This approach can also be used to examine the role of additional rare variants identified in centenarians (Zhengdong, *et al*. accepted for publication in Nature Metabolism).

## Methods

### Animals

Mice were housed in an SPF facility at the University of Washington under a 12-hour light and 12-hour dark cycle with room temperature of 25°C ± 4. Reverse osmosis water and irradiated food (Picolab Rodent Diet 20, 5053) were supplied. All studies were approved by the University of Washington IACUC. Mice were also kept in an SPF facility at the University of Minnesota under similar conditions. Mutant *Igfr1* (FVB-Tg (Ckm-IGF1R*K1003R)1Dlr/J) mice (*Igf1r*^*tg*^) were obtained from the Jackson Laboratory (Stock #016618). The animals harbor a K1003R mutation that inactivates the ATP-binding site in the enzyme, preventing autophosphorylation and phosphorylation of substrates ERK and AKT. Expression is driven by the creatine kinase muscle-specific promoter (Ckmm) and there are numerous copies of the transgene leading to strong expression of the mutant receptor in skeletal muscle and much lower expression in the heart [5]. These mice were crossed with inbred FVB *Ercc1*^+/Δ^ mice. Then *Igf1r*^*tg*^; *Ercc1*^+/Δ^ progeny further crossed with inbred C57BL/6 *Ercc1*^+/−^ mice to generate F1 *Igf1r*^*tg*^; *Ercc1*^−/Δ^ experimental mice. Breeding, health span and lifespan studies were approved by the UMN IACUC.

### CRISPR construct design

The human *IGF1R* gene encodes a mature protein with a length of 1367 amino acids (aa) and the mouse *Igf1r* gene encodes mature protein with a length of 1369 aa. Human and mouse IGF1R mature proteins result from cleavage of a 30-aa N-terminal signal peptide (Supplementary Figure 1) and amino acid numbering for IGFR is based on the mature protein [3]. A single-guide RNA (sgRNA) for *Streptococcus pyogenes* Cas9 (SpCas9) was used to mediate editing of the murine *Igf1r* gene. We submitted the nucleotide sequence containing *Igf1r* exon 6 to the Broad Institute Genetic Perturbation Platform (GPP) web portal [6–8]. We identified the sgRNA^*Igf1r*^ sequence ACCUGACGGUCAGGUUCCGG as a candidate for editing the part of exon 6 in the *Igf1r* gene that needs to be modified for the generation of *IGF1R*^hR407H^ mice at the mouse 408 residue position. Because of the different lengths of mouse and human IGF1R, the mouse arginine at position 408 corresponds to the human arginine at position 407 (Supplementary Figure 1). We used a 123-bp synthetic single-stranded DNA oligo donor (IDT, San Jose CA) that was based on the wild-type *Igf1r* sequence and contained two mutations that would result in destruction of the PAM sequence for sgRNA^*Igf1r*^ (TGG > TGA, synonymous mutation), and an amino-acid change from R to H (c.CGG > CAC, p.R408H).

### Generation and sequencing of pups

C57BL/6 female mice, 3–4 weeks of age, were super-ovulated by intraperitoneal injection of 5 IU of PMSG, followed by intraperitoneal injection of 5 IU hCG 48 hours later. Super-ovulated females were mated with adult C57BL/6 stud males and euthanized the next morning. Zygotes were harvested from the oviducts in M2 solution. Ribonucleoprotein (RNP) complex was prepared with 2 μM SpCas9 mRNA (Sigma) and 2 μM of sgRNA against *Igf1r* exon 6 (Synthego, Menlo Park CA) and incubated for 15 min at room temperature. Two batches of 37 embryos were electroporated with the RNP complex and 10 μM of single-stranded DNA oligo donor (IDT) in electroporation buffer [9] using a Bio-Rad (Hercules CA) electroporator (GenePulser Xcell: 30 V, 10 ms, 4 pulses) and cultured in KSOM media for 24 hrs. Twenty-nine 2-cell embryos were surgically transferred into the oviduct of two pseudopregnant mice (14+15). Three pups were born, and DNA was isolated three weeks later from tail tips using DNeasy extraction kit (Qiagen, Germantown MD). Nested PCR was performed around *Igf1r* sgRNA target site (oligo F: 5’ CCAGTAAGCAAGCAAACCCCT 3’ and oligo R: 5’ CACGGATGCCAAAGACTGTTA 3’). The PCR products were purified using EXO-SAP enzyme (ThermoFisher Scientific, Waltham MA) and sent for Sanger sequencing analysis (through Genewiz, Seattle WA) to check the genotyping of the mice.

### Genotyping

Briefly, tail biopsies from 8 to 10-day old pups were digested overnight at 37°C in tissue lysis buffer containing 10 mM Tris, 5 mM EDTA, 100 mM NaCl, 1% SDS and 50μg/ml proteinase K. The CRISPR nuclease targeted regions were PCR amplified with high-fidelity polymerase (Platinum Taq DNA Polymerase High Fidelity, Invitrogen). To detect gene edition, locus specific primers pair (oligo F: 5’ CAAGCACATTGTCCATACCTG 3’ and oligo R: 5’CACGGATGCCAAAGACTGTTA 3’) were used. PCR conditions were as follows: 35 cycles at 95°C for 30 s, 57°C for 30 s, and 72°C for 30 s. PCR products from wildtype and transgenic mice were subjected to TFiI restriction endonuclease to confirm the presence of the desired mutation. Restriction digestion was carried out at 65°C for 30 min, followed by gel electrophoresis for product size visualization on a 2% agarose gel. UMN mice were genotyped by TransnetYX (Cordova, TN).

### Healthspan and lifespan analysis of progeria mice

*Igf1r*^*tg*^; *Ercc1*^−/Δ^ and *Ercc1*^−/Δ^ mice were monitored for changes in health status twice per week beginning at weaning. The animals were monitored for symptoms listed in Table 1 and scored. Lifespan was measured by scoring deaths or terminal condition requiring euthanasia.

### Enzyme-linked immunosorbent assay (ELISA)

An ELISA kit was used to determine the level of IGF1 in the serum of mice (R&D System, UK) according to the manufacturer’s protocol. Briefly, serum samples from 6-month-old female mice were added into each well of a pre-coated ELISA plate and incubated at room temperature for 120 min. After that, wells were washed 5 times and an HRP-conjugated antibody was added, followed by incubation for 120 min. Next, wells were washed 5 times, substrate solution was added and incubated for 30 min. Then, stop solution was added and the absorbance at 450 nm was determined using a microplate reader (Synergy H1, BioTek, Winooski, VT, USA).

### Immunoblots

Heart, kidney, pancreas, and skeletal muscle were isolated from 6-month old mice and snap frozen at −80 °C. Protein extraction was carried out using T-PER^™^ Tissue Protein Extraction Reagent (ThermoFisher Scientific, Waltham MA) with Halt^™^ Protease Inhibitor Cocktail (ThermoFisher Scientific, Waltham MA) and Halt^™^ Phosphatase Inhibitor Cocktail (ThermoFisher Scientific, Waltham MA). Protein concentration of each sample was determined by BCA Protein Assay Reagent (Pierce, Rockford IL). Standard electrophoresis was performed. Blocking and antibodies were prepared in filtered 5% BSA. The blots were developed using Clarity Max Western ECL Substrate (Bio-Rad, Hercules CA) and were scanned using Azure Imager C300 (Azure Biosystems, Dublin CA). The densitometric analysis was conducted to quantify the Western blot immunoreactivity using Image J. The primary antibodies used were IGF-I Receptor β (1:1000, #9750S), Phospho-IGF-I Receptor β (1:1000, #3918S), Akt (pan) (1:1000, #4691S), Phospho-Akt (1:2000, #4060S), and Actin (pan) (1:1000, #4968S). All primary antibodies were obtained from Cell Signaling Technology (Danvers MA). The secondary antibody used was goat anti-rabbit IgG-HRP (1:4000; Santa Cruz Biotechnology, Santa Cruz CA).

### Statistical analysis

All the data are presented as mean ± standard error of mean (SEM) or ± standard deviation (SD). Statistical comparisons were performed with the independent t-test. The criterion for statistical significance was chosen to be p<0.05. For animal studies, mice harboring a mutation in *Igf1r* or not were compared in a progeroid *Ercc1*^-/ Δ^ background using an unpaired Student’s t-test.

## Results and discussion

### The generation of IGF1R^hR407H^ mice using CRIPSR/Cas9 gene editing

**Figure 1.**
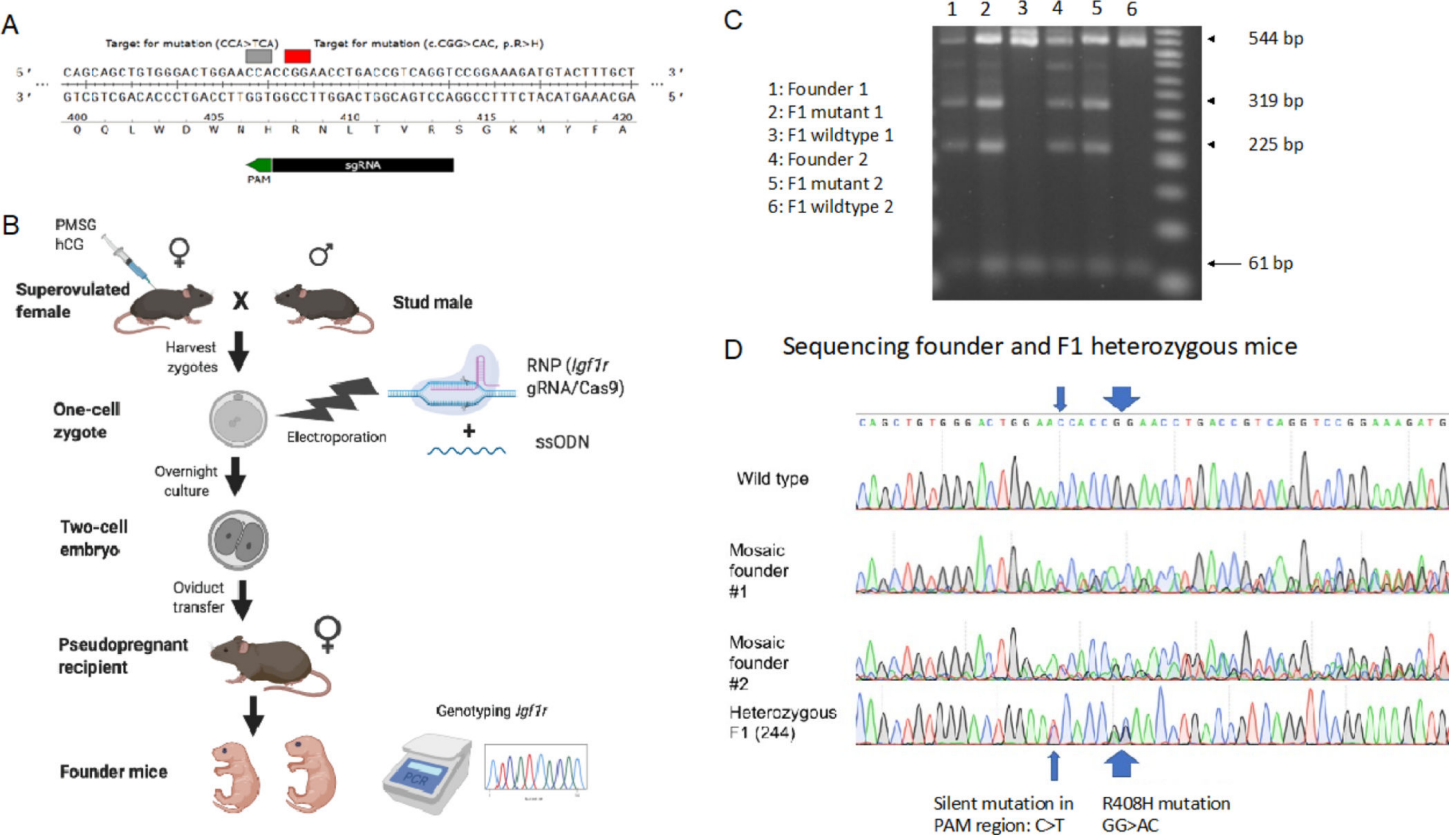
IGF1R^hR407H^ mice were generated using CRIPSR/Cas9 editing. A. Schematic of the *Igf1r* targeting strategy. The sgRNA^*Igf1*^ is indicated in black and its PAM sequence in green. The mutation introduced in amino acid 408 (R>H: CGG>CAC) and the silent mutation in the PAM sequence (CCA>TCA) are indicated in red and grey, respectively. **B.** Experimental workflow. Female mice were super-ovulated using PMSG and hCG and housed with stud males for breeding. One-cell zygote embryos were collected and electroporated with sgRNA^*Igf1*^/Cas9 RNP complex and ssODN. The next day, embryos were transferred to pseudo-pregnant recipient to generate edited mice. The resulting pups were genotyped by PCR and Sanger sequencing analysis. **C.** Electrophoresis gel showing the PCR fragments pattern from two founders and their wildtype and heterozygous F1 IGF1R^hR407H^ mice after digestion at the Tfil restriction sites. **D.** Sanger sequencing analysis of exon 6 of *Igf1r* in F0 founder mice and one of the heterozygous F1 mice (pup 244)

We generated a mouse line that replicates the human IGF1R R407H longevity-related variant observed in Ashkenazi Jewish centenarians using CRIPSR/Cas9 technology to generate an R408H substitution in the mouse *Igf1r* gene. The first step was electroporation of the RNP consisting of the sgRNA^*Igf1r*^ and SpCas9 mRNA with a 123-bp single stranded oligonucleotide donor template (ssODN) into one-cell mouse zygotes. We designed the ssODN having three base mismatches to the genomic sequence to introduce the CGG > CAC substitution that changes the Arg at mouse position 408 to His by HDR, and to direct the synonymous mutation (TGG > TGA) at the Protospacer Adjacent Motif (PAM) sequence to destroy the PAM with introduction of a Tfil restriction site at the same time for later mouse genotyping (Figure 1A). After electroporation and overnight culture, the two-cell embryos were surgically transferred into the oviduct of pseudopregnant mice. The founder pups were genotyped and sequenced for the three nucleotide substitutions (Figure 1B). The line was designated as h(human)R407H (IGF1R^hR407H^) to show that at mouse position 408, the human R407H variant was correctly represented (Supplementary Figure 1).

Three pups were born and screened for the presence of the hR407H mutation using PCR amplification and DNA sequencing. Two of the pups had the hR407H mutation present with the desired mutation at the PAM region in their genome while the third pup had only random insertions/deletions. In all 3 mice, Sanger sequencing showed that additional sequences were present in the DNA extracted from the tails, revealing a high degree of mosaicism (Figure 1D), which has been commonly reported in CRISPR edited mice [10]. We bred the two F0 founder mice with C57BL/6 wildtype mice and assessed the germline transmission of the IGF1R hR407H allele. We genotyped the F0 founders and F1 mice using PCR and restriction endonuclease digestion at the TfiI sites to confirm the presence of the desired mutation. Since the amplified region from wildtype mice had only one TfiI site whereas heterozygous IGF1R hR407H mice had two, the product sizes of wildtype mice samples were 61 base pairs (bps) and 544 bps, whereas the product sizes from IGF1R hR407H mice were 61 bps, 225 bps, 319 bps, and 544 bps after the restriction digestion (Figure 1C). At the end, 9 out of 13 F1 pups obtained from both founders were heterozygous for the hR407H mutation, with further sequencing data demonstrating the desired mutations with no off-target editing, indicating high efficiency and accuracy of our design. (Figure 1C, D).

### Expression levels of IGF1R were altered in female mice with the IGF1R^hR407H^ genotype

**Figure 2.**
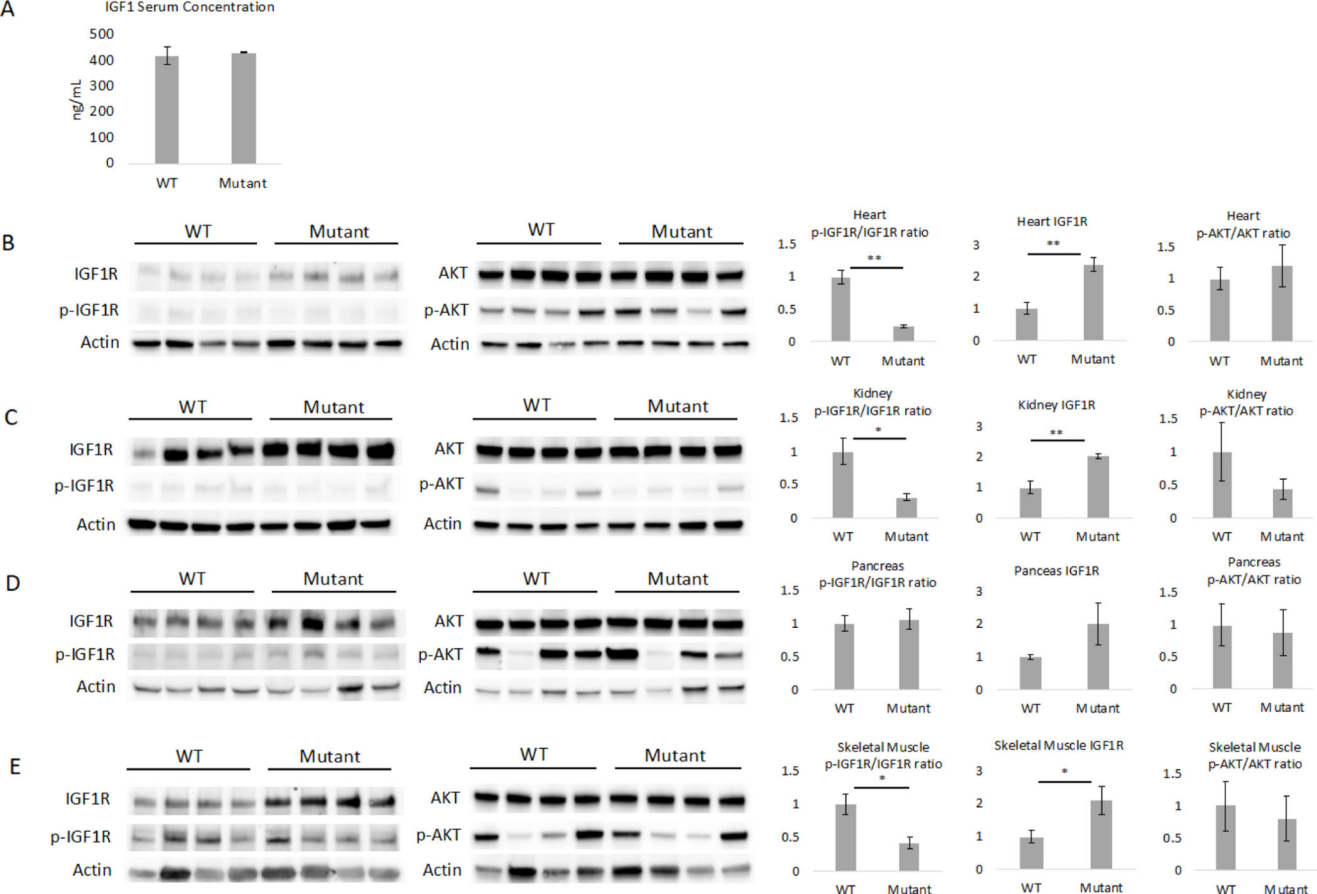
Female mice with the IGF1R^hR407H^ genotype showed alterations in IGF1R expression levels. **A.** Serum IGF1 levels in female WT and IGF1R^hR407H^ (mutant) mice at 6 months of age (n=4–5). **B-E.** Western blotting was performed to measure the phosphorylation levels of IGF1R, AKT and the total IGF1R expression in heart **(B)**, kidney **(C)**, pancreas **(D)**, and skeletal muscle **(E)** tissues (n=4 mice per genotype). The phosphorylated to total protein ratios and IGF1R total protein expression, as determined by densitometry, are shown on the right (B-E). IGF1R expression levels were normalized to each individual actin. All data represent the mean ± SEM. *p < 0.05, **p < 0.01, statistically significant difference between WT and IGF1R^hR407H^ mice

After successfully generating the F1 heterozygous IGF1R^hR407H^ genotype, we searched for alterations in IGF1R function. Since a previous study of R407H centenarian carriers showed a more robust impaired-IGF1 signaling phenotype in females but not in males [3], we first measured serum IGF1 levels in 6-month-old wildtype (WT) and IGF1R^hR407H^ female mice. We found there was no difference in the IGF1 serum concentration between WT and mutant female mice (Figure 2A), in contrast with the results seen in female centenarian offspring whose serum IGF1 concentrations were significantly higher than those of controls. To further investigate changes in IGF1R signaling in mutant female mice, we analyzed the phosphorylation of IGF1R and AKT in heart, kidney, pancreas, and skeletal muscle. We found a significant decrease In IGF1R phosphorylation levels in mutant mice compared to WT mice in heart, kidney, and skeletal muscle, but not in the pancreas (Figure 2B–E). Interestingly, there was a two-fold increase in the IGF1R expression in IGF1R^hR407H^ mice in all the tissues, with a significant increase in heart, kidney, and skeletal muscle (Figure 2B–E). However, no significant decrease in the phosphorylation level of AKT was observed in any of the tissues, although there was a downward trend observed in the kidney (Figure 2B–E).

The decreased IGF1 signaling level in IGF1R^hR407H^ female mice helps to verify the integrity of the mouse line and relevance as a model for the human longevity-related mutation. However, our IGF1R^hR407H^ mice show a discrepancy from the human centenarian offspring carriers in terms of no significant increase in serum IGF1 level and no significant decrease in IGF1R level in the tissues [3]. In fact, the significant increase in IGF1R expression in multiple organs suggests a potential positive feedback mechanism between the IGF1R^hR407H^ variant and expression of IGF1R independent of IGF1 levels in mutant mice. This might indicate differences between human and mice in terms of how the IGF1R^hR407H^ variant induces a feedback mechanism to compensate for the reduction of IGF1 signaling level. The failure to observe any difference in the downstream signaling of AKT in IGF1R^hR407H^ mice could be related to the young age of the mice. More significant changes may be seen when older animals become available for study. In addition, actual studies on signaling in response to an IGF1 bolus would help to establish physiological proof that IGF1R signaling is downregulated in IGF1R^hR407H^ mice.

### Overexpression of kinase-dead IGF1R in skeletal muscle extends the healthspan and median lifespan of progeroid *Ercc1*^*−/Δ*^ mice

Since we found a significant decrease in IGF1R phosphorylation level in skeletal muscle of IGF1R^hR407H^ mice, we wanted to determine if reduced IGF1R signaling specifically in skeletal muscle could promote healthspan and lifespan. To facilitate these experiments, the *Ercc1*^−/Δ^, which model a human progeroid syndrome and have an accelerated aging phenotype [11] was utilized. The *Ercc1*^−/Δ^ mice were crossed with mice expressing in skeletal muscle an IGF1R with a K1003R mutation that inactivates the ATP-binding site in the enzyme, preventing autophosphorylation and phosphorylation of substrates in skeletal muscle upon ligand binding.

**Figure 3.**
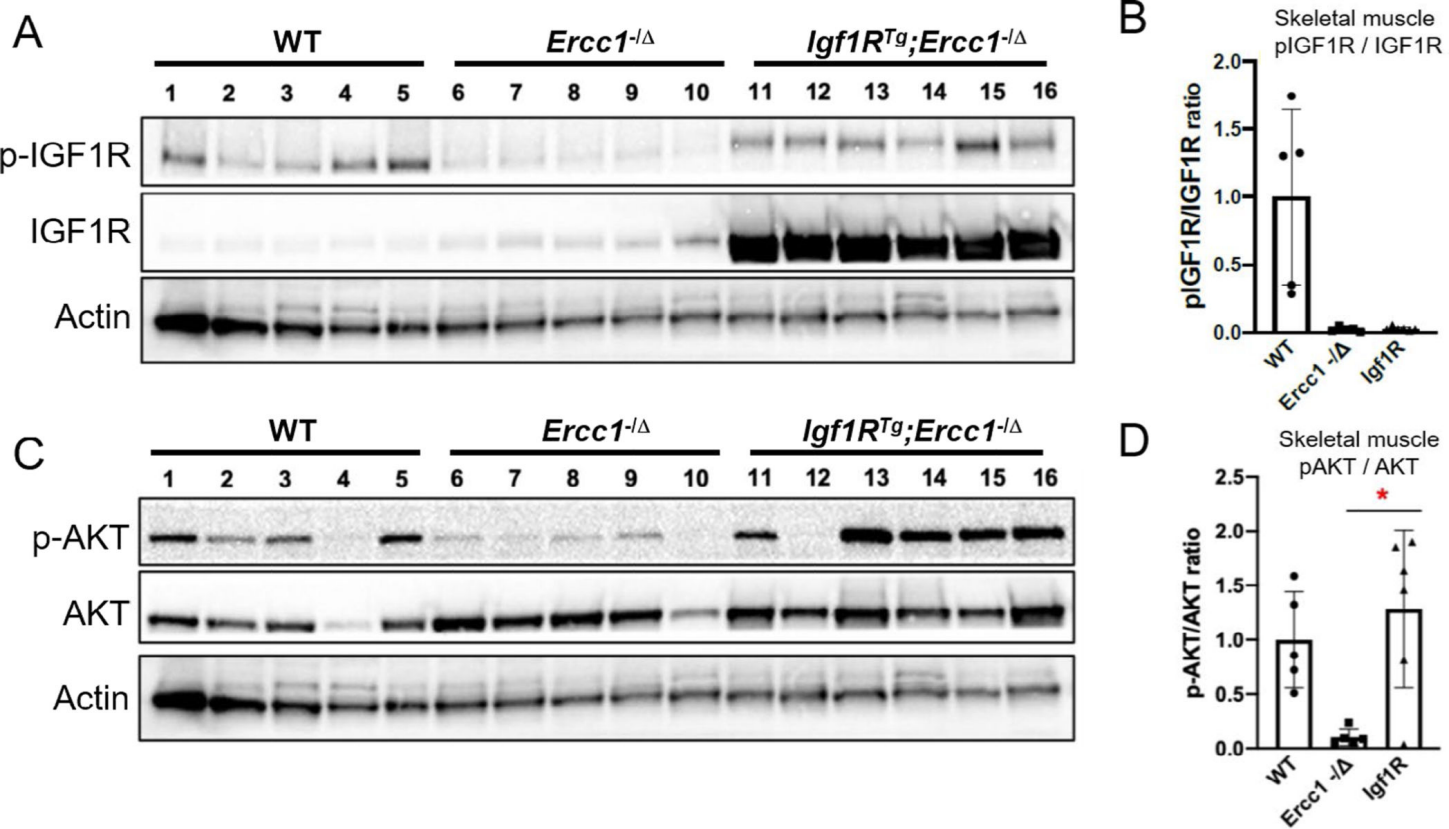
Overexpression of mutant IGF1R in progeroid ***Ercc1***^**−/Δ**^ mice. *Ercc1*^+/−^; *Igf1R*^Tg^ inbred C57BL/6 mice were crossed with inbred FVB/n *Ercc1*^+/**Δ**^ mice to produce progeroid mice harboring the *Igf1R* transgene driving over expression of mutant IGF1R in differentiated myocytes from the creatinine-kinase muscle-specific promoter. **A.** Immunoblot detection of IGF1R expression and the auto-phosphorylated receptor in skeletal muscle of the mutant animals compared to controls. **B.** Quantitation of the immunoblot data using ImageJ. Values are standardized using Actin as a loading control and reported is the ratio of the phosphorylated receptor to non-phosphorylated. **C.** Immunoblot detection of AKT and phosphorylated AKT in skeletal muscle of the same animals. **D.** Quantitation of the immunoblot data using ImageJ. Values are standardized using Actin as a loading control and reported is the ratio of the phosphorylated receptor to non-phosphorylated. Shown are the mean +/− S.D. Paired t-test, *p<0.05

Immunoblot analysis of protein isolates prepared from quadricep muscle indicates strong overexpression of IGF1R in skeletal muscle of the transgenic mice (Figure 3A–B). Phosphorylation of IGF1R is already low in *Ercc1*^−/Δ^ mice consistent with suppression of the IGF1 axis, previously reported in *Ercc1*^−/−^ mouse tissues. AKT expression was unchanged in skeletal muscle of the transgenic mice (Figure 3C–D). However, phosphorylation was increased, albeit inconsistently, in the skeletal muscle of *Igf1R*^Tg^; *Ercc1*^−/Δ^ mice compared to *Ercc1*^−/Δ^, as was previously reported for *Igf1R*^Tg^ mice.

**Figure 4.**
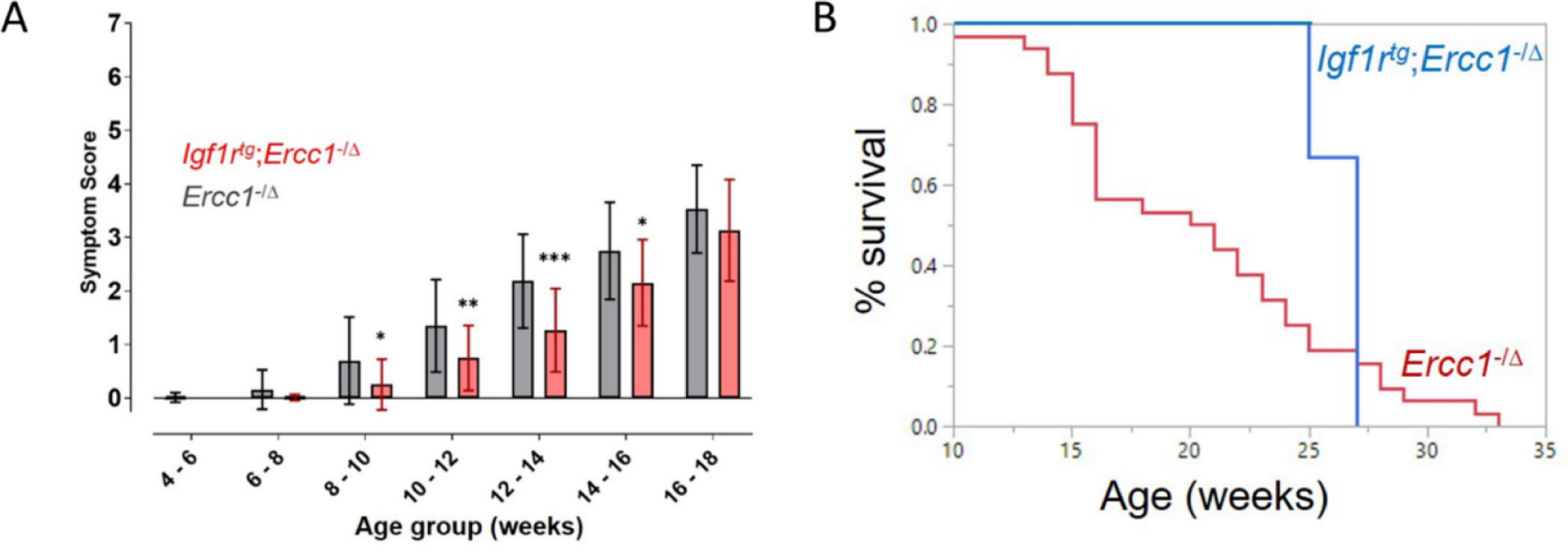
Healthspan and median lifespan are extended in progeroid ***Ercc1***^***−/Δ***^ mice overexpressing kinase-dead IGF1R in skeletal muscle. **A.** The onset and severity of kyphosis, ataxia, impaired ambulation, dystonia, tremors and poor body condition were measured twice per week as described in methods

A cohort of *Igf1r*^*tg*^; *Ercc1*^−/Δ^ mice were bred and their health monitored across their lifespan and compared to *Ercc1*^−/Δ^ mice. As previously reported [5], mice harboring the *Igf1R* mutant transgene had significantly reduced body weight compared to littermate controls (Supplemental Figure 2). The *Igf1r*^*Tg*^; *Ercc1*^−/Δ^ mice showed a significant delay in the onset of age-related symptoms characteristic of progeroid *Ercc1*^−/Δ^ mice (Figure 4A). *Igf1r*^*tg*^; *Ercc1*^−/Δ^ mice also showed a dramatic extension of median lifespan compared to progeroid *Ercc1*^−/Δ^ mice (Figure 4B), consistent with an extension of healthy aging. As a result, depletion of IGF1R signaling specifically in skeletal muscle can increase the health span and medium lifespan of progeroid mice, providing the rationale for conducting lifespan and healthspan studies in IGF1R^hR407H^ mice.

## Conclusions

We have generated a novel IGF1R^hR407H^ mouse line that carries a longevity-related variant enriched in human centenarians. This mouse line serves as a prototype for studying genetic-related longevity in humans and furthers the field of longevity research to promote healthy aging. Lifespan studies now are needed to confirm the longevity of IGF1R^hR407H^ mice and to help show that the variant plays a role in human longevity. The mouse line serves as a platform for studying longevity associated genetic variants with high translational value for developing drugs that can target these variants to promote healthy aging. Finally, the customized CRISPR editing approach represents a prototype for generating additional longevity associated gene variant mouse lines to study relevance in aging and longevity.

## Supplementary Material

supplementary material

## Figures and Tables

**Table 1.** Symptoms and scoring severity to calculate an aging score, which is a measure of health span

Symptoms	Test	Score	Description
Kyphosis	Inspect the mouse for curvature of the spine or hunched posture. Run your fingers down both sides of the spine to detect abnormalities.	0	Absent
		0.5	Mild curvature
		1	Clear evidence of hunched posture
Ataxia	Observe gait and stance on a smooth surface	0	Absent
		0.5	Occasional non-toe spread in stance and gait
		1	Ataxia postures present (no toe spread, foot dragging)
Gait disorders	Observe the freely moving animal to detect abnormalities such as hopping, wobbling, circling, wide stance and weakness.	0	No abnormality
		0.5	Abnormal gait but animal can still walk
		1	Marked abnormality, impairs ability to move
Dystonia	Hold Mouse by tail to for 20 secs and observe hind limb splay	0	No motor abnormalities
		0.25	One or both legs are drawn in within 20 secs
		0.5	One or both legs are drawn in within 10 secs
		0.75	One leg Is drawn in immediately
		1	Both legs are drawn in immediately
Tremor	Observe the freely moving animal to detect tremor, both at rest and when the animal is trying to climb up an incline.	0	No tremor
		0.5	Slight tremor
		1	Marked tremor
Body condition	Place mouse on flat surface, hold tail base and manually assess the flesh/fat that covers the sacroiliac region (back and pubic bones).	0	Bones palpable. not prominent
		0.5	Bones prominent or barely felt
		1	Bones very prominent or not felt due to obesity
