# Supplementary material for: Development of an IGF1R longevity variant mouse line using CRISPR/Cas9 genome editing
Source: Trends Biomed Res. Author manuscript; Available in PMC 2023 Apr 26. (PMC10131096; doi:10.15761/jtbr.1000121)
Supplement: supplementary material [file NIHMS1756003-supplement-supplementary_material.pdf]

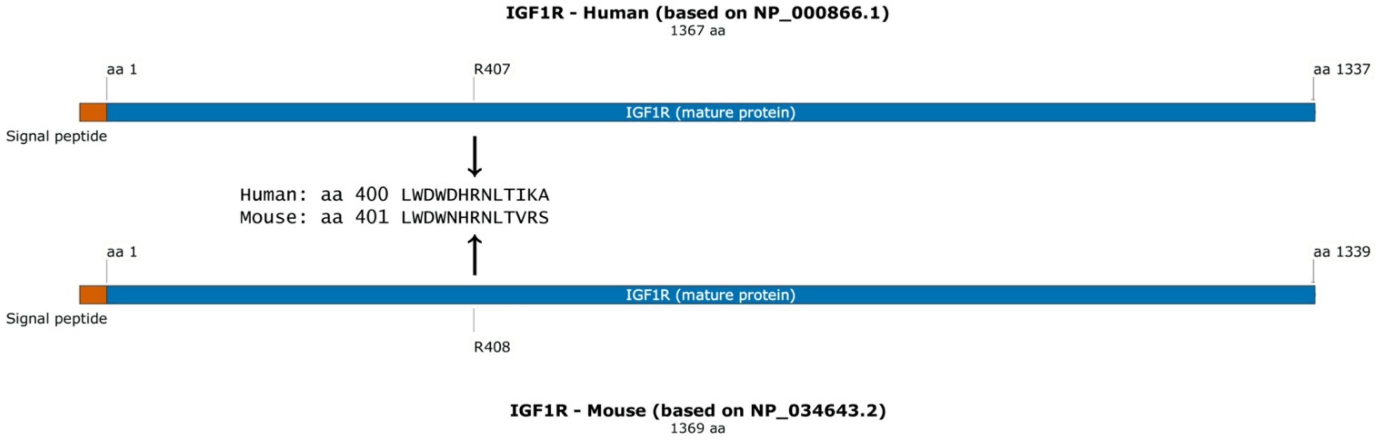

Supplemental Figure 1. Schematic of human and mouse IGF1R protein. A protein schematic showing the different lengths of mouse and human IGF1R, with the mouse arginine at position 408 corresponding to the human arginine at position 407

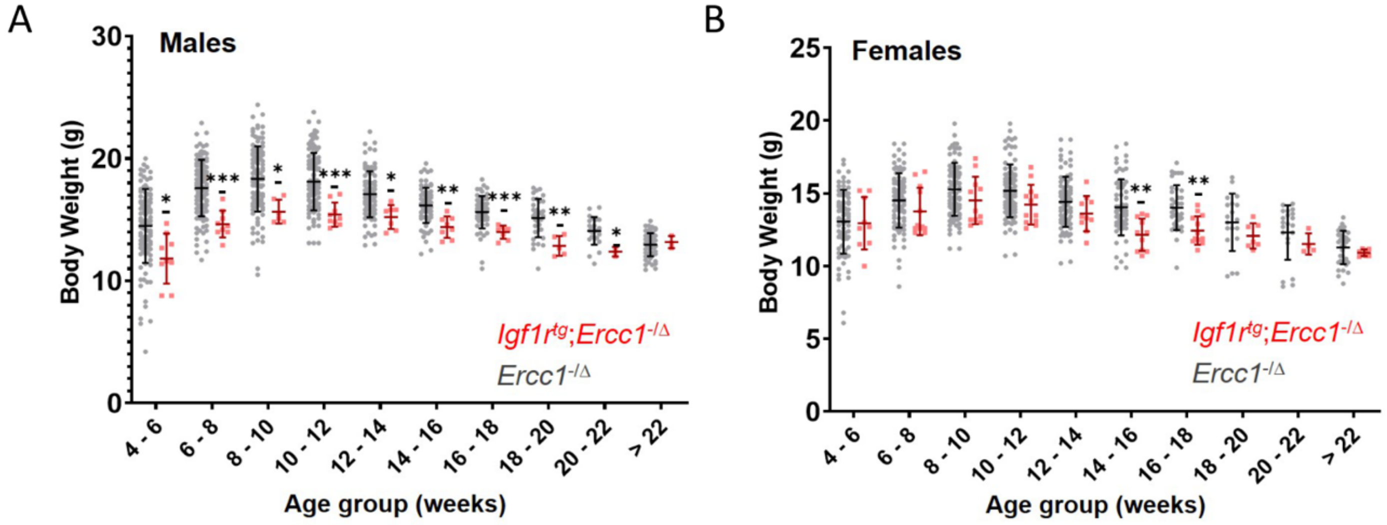

Supplemental Figure 2. Body weights of male and female *Igf1r^tg^*; *Ercc1*$^{-/\Delta}$ and *Ercc1*$^{-/\Delta}$ mice. Graphed are the body weights of individual males (A) and females (B) with the mean and standard deviation for each group. N $\geq$ 4 per group. p values were determined by an unpaired Student's t-test *p<0.05, **p<0.01, ***p<0.001

Copyright: ©2020 Dou Y. This is an open-access article distributed under the terms of the Creative Commons Attribution License, which permits unrestricted use, distribution, and reproduction in any medium, provided the original author and source are credited.
